# Early MRI imaging and follow-up study in cerebral amyloid angiopathy

**DOI:** 10.1515/med-2021-0212

**Published:** 2021-02-02

**Authors:** Shan-chun Zhang, Jian-jun Jia, Heng-li Zhao, Bo Zhou, Wei Wang, Xiang-hui Lu, Hao Wang, Zhen-fu Wang, Wei-ping Wu

**Affiliations:** Geriatric Neurological Department of the Second Medical Centre and National Clinical Research Center of Geriatric Disease, Chinese People’s Liberation Army General Hospital, Beijing, 100853, China; Geriatric Cardiological Department of the Second Medical Centre and National Clinical Research Center of Geriatric Disease, Chinese People’s Liberation Army General Hospital, Beijing, 100853, China

**Keywords:** cerebral amyloid angiopathy, leukoaraiosis, cerebral micro bleeding, superficial siderosis, susceptibility weighted imaging, intracranial hemorrhage

## Abstract

**Aim:**

To study the imaging features of leukoaraiosis (LA) and hemorrhage in cerebral amyloid angiopathy (CAA) patients.

**Methods:**

The earliest MRI images of probable CAA patients and non-CAA patients were collected. The characteristics of LA in the two groups were analyzed. Cerebral micro bleeding (CMB), superficial siderosis (SS), and intracranial hemorrhage (ICH) were recorded in the follow-up study. The space relationship between CMB or SS and ICH was assessed.

**Results:**

We found that 10/21 (47.6%) patients had occipital prominent LA and 14/21 (66.7%) patients had subcortical punctate LA before the ICH, which was higher than that of the ones in the control group (*p* = 0.015 and 0.038, respectively). The recurrence rate of ICH was 100% (3/3) in patients with diffuse SS and 36.4% (4/11) in patients without. The recurrence rate of ICH was 60% (3/5) in patients with multiple-lobe CMBs and 44.4% (4/9) in those without. The location of the ICH and CMB was inconsistent. ICH occurred in the ipsilateral cerebral hemisphere of SS in three patients with diffuse SS.

**Conclusion:**

LA, diffuse SS, and multiple-lobe CMBs are important imaging characteristics of CAA, which may help make early diagnosis and predict the recurrence of ICH.

## 1 Introduction

Recurrent lobar hemorrhage is a common clinical manifestation of cerebral amyloid angiopathy (CAA). However, intracranial hemorrhage (ICH) occurs mostly in the late stage of CAA, which hinders the early diagnosis of CAA.

Leukoaraiosis (LA) is a common imaging manifestation of cerebral small vascular disease. Different distribution of LA in the anterior and posterior horns of the lateral ventricle has been observed in different populations. In the normal elderly population, lesions in the frontal horn were predominantly observed [1], whereas in patients with CAA-related ICH, lesions in the occipital horn were mainly found [2]. However, little clinical follow-up study focuses on leukoencephalopathy (LA) before ICH in probable CAA patients.

Cerebral micro bleeding (CMB) and superficial siderosis (SS) detected in magnetic susceptibility weighted imaging (SWI) are early hemorrhagic features before ICH. Recent studies show cerebral CMB or SS may relate to the recurrence of ICH [3,4,5]. However, the relationship between cerebral CMB or SS and ICH is poorly understood.

In this study, we analyzed the distribution of LA before hemorrhage in CAA patients and non-CAA patients to help the early diagnosis. We also observed the location of CMB or SS and the recurrence of ICH to investigate the mechanism of hemorrhage.

## 2 Materials

### 2.1 Objects of the study

**Figure 1:**
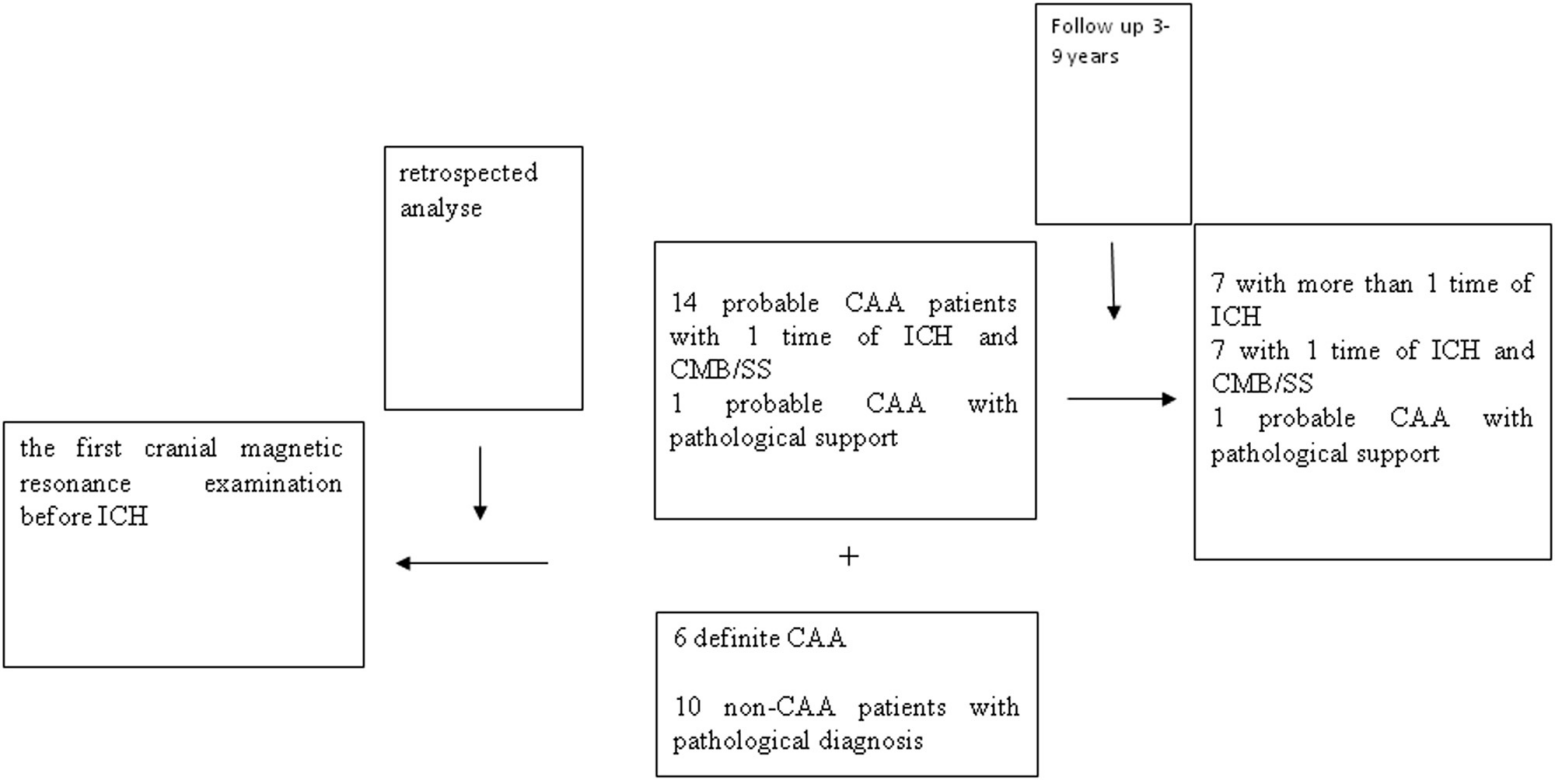
Flow diagram showing study enrollment and results.

Twenty-one cases of CAA patients aged 55 years and over with complete head magnetic imaging data (including T1 weighted phase, T2 weighted phase, and FLAIR phase) were selected, including seven pathologically supported CAA patients (autopsy 6, biopsy 1) and 14 probable CAA patients. The diagnostic criteria for CAA patients used in our study were consistent with the Boston criteria revised in 2010 [6]. The control group consisted of ten non-CAA patients with pathological diagnosis. Patients with acute cerebrovascular disease, brain tumors, or extensive severe periventricular LA were excluded. Their diagnoses are: four with pneumonia, four with coronary heart disease, one with lung cancer, and one with prostatic cancer (Figure 1). The study design was approved by the Ethics Committee of PLA General Hospital and performed in accordance with the Declaration of Helsinki.

### 2.2 Study methods

#### 2.2.1 Measurement of LA

The brain MRI was performed with GE 3T and 1.5T scanners. MRI sequences included the spin echo axial T1WI, T2WI, and FLAIR. The scanning parameters were T1WI (TR 1,875 ms, TE 24 ms, TI 750 ms, thickness 6 mm, gap 0.5 mm, and matrix 288 × 256), T2WI (TR 5,500 ms, TE 127 ms, thickness 6 mm, gap 0.5 mm, and matrix 352 × 352), and FLAIR (TR 8,400 ms, TE 120 ms, TI 2,100 ms, thickness 6 mm, gap 0.5 mm, and matrix 288 × 192). The degree of LA was assessed by the method of Fazekas et al., and separate measurements were performed of the periventricular and deep white matter in FLAIR [7]. The scoring standard for periventricular white matter was as follows: 0 point: none; 1 point: cap or thin line; 2 points: smooth “halo” sample; 3 points: irregular protrusion to deep white matter. The scoring standard for deep white matter was as follows: 0 point: none; 1 point: spotted; 2 points: starting fusion; 3 points: large fusion. According to this method, the white matter in the anterior and posterior horns of lateral ventricles, deep white matter, and subcortical white matter were scored separately. The total scores of the frontal and occipital lobes were calculated separately by adding scores of the three parts together. The difference between the two was recorded as the fronto-occipital value (FO value). FO value >2 was frontal prominent and FO value <−2 was occipital prominent [8]. The distribution of subcortical white matter was divided into four types according to Charidimou’s category in 2016: multiple subcortical punctate LA, linear LA around the basal ganglia, posterior subcortical large LA, and anterior subcortical large LA [9].

#### 2.2.2 SWI measurement method

The SWI scanning parameters were repetition time/echo time = 27/20 ms, flip angle = 15°, acceptance bandwidth = 120, field of view = 22 cm, and matrix = 256 × 220, single excitation. CMB refers to the dot-like low signal with a diameter of 2–5 mm on the SWI sequence, and no edema was observed around. SS refers to the linear low signal distributed along the gyrus of SWI sequence. Focal SS was limited to three or less cerebral gyri, whereas diffuse SS affected more than three cerebral gyri. CMB was divided into deep white matter type and subcortical type according to its location, and the number of CMB lesions and cortical SS in each part of brain was recorded. All imaging features were assessed after discussion by the team without knowing the history of the disease, and then reviewed by an imaging specialist. Radiologists and neurologists were unaware of the clinical diagnosis.

#### 2.2.3 Statistical method

Measurement data were expressed by mean ± standard deviation, and enumeration data are expressed by numbers. Fisher’s test was used to compare the incidence of hemorrhage in different lobe. For the LA in CAA patients and non-CAA patients, SPSS 17.0 software was used for the statistical analysis, and Fisher’s exact probability test was used to compare the rates.

## 3 Results

1. All 21 patients suffered from lobar hemorrhage, and most of the cases included large foci, lobulated, miliary, petechiae, and spindle hemorrhage. Of them, eight cases had multiple lobar hemorrhages, three had convex subarachnoid hemorrhage, and two with ICH breaking into the ventricle. Twelve cases with occipital lobar hemorrhage (34.3%) and ten cases with frontal lobar hemorrhage (28.6%), as well as eight cases (22.9%) with temporal lobar hemorrhage and five cases (14.3%) with parietal lobar hemorrhage. No statistical difference was observed among the groups.
2. Table 1 showed the distribution of LA in CAA patients and non-CAA patients. Of the 21 patients, 10 (47.6%) had occipital prominent LA before ICH, a percentage that was higher than that in the control group (*p* = 0.015). In addition, 14/21 (66.7%) patients had subcortical punctate LA before ICH, which was also higher than that of the ones in the control group (*p* = 0.038).
3. We observed the position of CMB or SS and the recurrence of ICH in seven patients with more than one time of ICH. The general clinical features are presented in Table 2. Of these seven cases, three were with diffuse SS, three were found to have multiple-lobe CMBs (>3), and three cases were found to have only 1–2 lobe CMB lesions. Two cases were accompanied by CMB in the basal ganglia. The position of CMB or SS and recurrence of ICH are presented in Table 3. Of the seven patients with one time of ICH, one case had focal SS, two had multiple-lobe CMB lesions, two had 1–2 lobe CMB lesions, and two had no CMB.
  The recurrence rate of ICH was 100% (3/3) in patients with diffuse SS and 36.4% (4/11) in patients without diffuse SS. The recurrence rate of ICH was 60% (3/5) in patients with multiple-lobe CMBs and 44.4% (4/9) in those without multiple-lobe CMBs.
4. We observed the relationship between CMB or SS and the location of ICH or convex subarachnoid hemorrhage. We defined that the location of SS or CMB, which was consistent with that of ICH if CMB or SS was present within the scope of ICH. The location of ICH in eight patients was not consistent with CMB. Of the three patients with SS, two cases had ICH and one case had subarachnoid hemorrhage. The location of hemorrhage was inconsistent with that of previous SS, but it was in the ipsilateral cerebral hemisphere of SS.

**Table 1:** Summary of the general and clinical information of probable CAA patients with more than one time of ICH (*n* = 7)

Serial number	Diagnostic basis	Sex	Age	Hypertension	Diabetes mellitus
8	Two lobar hemorrhages	m	90	0	1
9	Two lobar hemorrhages	m	99	1	0
10	Two subarachnoid hemorrhages	m	81	1	0
12	Six lobar hemorrhages	m	86	0	0
13	Two lobar hemorrhages	m	84	1	0
14	One lobar hemorrhage + one convex subarachnoid hemorrhage	m	70	0	1
15	One lobar hemorrhage + one subarachnoid hemorrhage	m	84	0	0

Note: 0: no; 1: have m: male; f: female.

**Table 2:** Distribution of LA in CAA group and non-CAA group

	CAA group (*n* = 21)	Non-CAA group (*n* = 10)	*p*-value
Age	80.5 (70–99)	83.1 (76–88)	0.786
Hypertension (%)	13 (61.9%)	6 (60%)	N
FO value (−1 to 1)	10 (47.6%)	5 (50%)	0.901
FO value (<−2)	10 (47.6%)	1 (10%)	0.029
FO value (>2)	1 (4.8%)	4 (40%)	0.015
Subcortical punctate LA	14 (66.7%)	3 (30%)	0.038

**Table 3:** The position of CMB or SS and recurrence of ICH in eight probable CAA patients with more than one time of hemorrhage (*n* = 7)

Serial number	Occipital lobe	Frontal lobe	Parietal lobe	Temporal lobe	Basal ganglia	Cerebellum	Location of ICH
8	1			3SS			Right temporal occipital lobe
9		1	1				Right temporal lobe
10	2		6 + 11SS	2	1		Subarachnoid space
12	1SS	3SS	1	1	1		Right occipital lobe
13	10			2		2	Right temporal lobe
14	2						Right occipital lobe
15		1		2			Right temporal lobe

**Figure 2:**
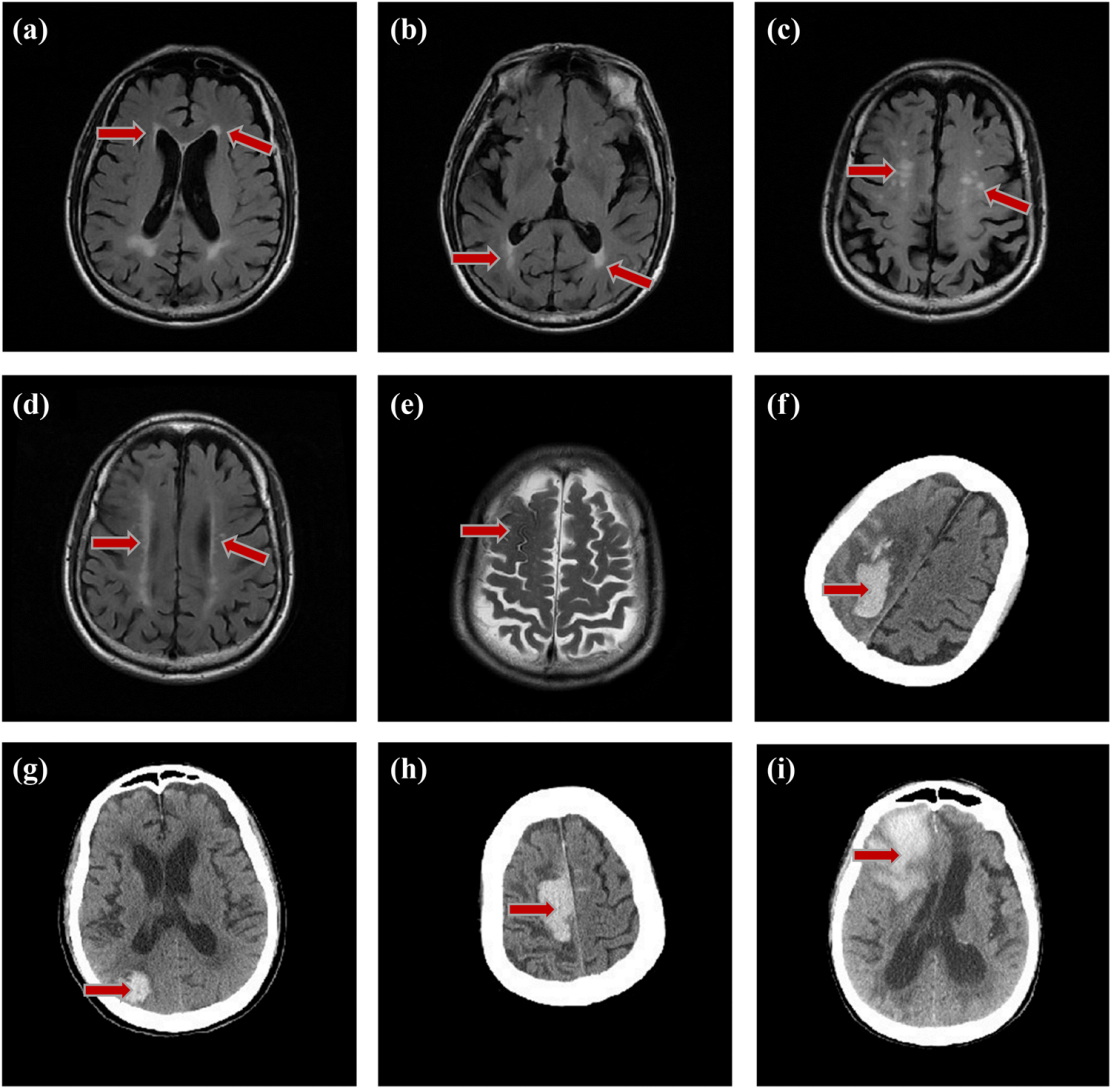
Distribution of early LA and hemorrhages in case 12. An 86-year-old male patient, occipital prominent LA and subcortical patchy distribution of white matter appeared 3 years before the ICH. (a) Score 1 in the frontal lobe white matter (periventricular 1, deep 0, juxtacortical 0). (b) Score 4 in the occipital lobe white matter (periventricular 3, deep 1, juxtacortical 0). (a and b) FO value = −3 occipital prominent LA. (c) Subcortical multiple punctate LA, without clear periventricular white matter damage. (d) The bilateral white matter lesions were asymmetric, with the right white matter lesions severer. (e) T2 shows old convex subarachnoid hemorrhage in August 2009. (f) Shows that ICH occurred in right parietal-occipital lobe in 2012. (g–i) CT shows recurrent ICH occurred in right occipital lobe, parietal lobe, and frontal lobe in 2014, 2016, and 2018, all on the same side of convex subarachnoid hemorrhage.

Below, we presented a follow-up of the imaging of LA, CMB, and ICH in a probable CAA patient (Figure 2). Before ICH, occipital prominent LA and subcortical LA were observed. LA occurred 3 years before ICH. The location of recurrent lobar hemorrhage was not consistent with CMB, but was in the ipsilateral cerebral hemisphere of the diffuse SS.

## 4 Discussion

### 4.1 Participants

Most of the studies on LA in CAA patients included patients with lobar CMB or probable CAA patients with ICH. The former only met the criteria of possible CAA. The latter had been in the late stage of the disease, and the white matter lesions became more severe, which could not reflect the characteristics of early LA. We selected probable CAA patients and retrospectively analyzed the first brain MRI before ICH, which could reflect the early LA in CAA patients. The control group included patients with no CAA changes in autopsy to exclude pre-clinical CAA patients.

### 4.2 Coexistence of white matter lesions and ICH in CAA patients

The present follow-up study showed a whole imaging progress from the early sign of LA, CMB or SS to the recurrence of hemorrhage in CAA patients. We found that posterior prominent white matter distribution and subcortical multiple punctate LA in CAA patients might occur before lobar hemorrhage, as shown in former studies [8,9,10].

Imaging studies suggested that there was a correlation between hemorrhagic lesions and non-hemorrhagic lesions in CAA patients [2], but few studies followed up with both white matter lesions and hemorrhagic imaging changes in CAA patients. Our study of early white matter lesions and hemorrhagic imaging in CAA patients could reflect the progress of pathological changes in CAA patients. First of all, we found that white matter changes could occur several years earlier than ICH, supporting that white matter lesions might be used as imaging markers before ICH. The consistency of hemorrhagic and non-hemorrhagic imaging manifestations supported the diagnosis of CAA, whereas a patient suffered only lobar hemorrhage without white matter lesions might not support CAA. Second, our follow-up study also noticed that bilateral white matter lesions could be asymmetrical and hemorrhages occur on the same side with severer LA, as shown in Figure 2, which supported the correlation between LA and ICH.

### 4.3 Recurrence of ICH or convex subarachnoid hemorrhage can be asymmetrical in CAA patients with SS

SS presence and extent are the most important MRI prognostic risk factors for lobar ICH recurrence [11,12,13,14,15], and diffuse SS is regarded as a risk factor of recurrent hemorrhage [16,17]. The deposition of amyloid protein may be the pathological basis of the severer white matter lesion, SS, and ICH [8,10,18]. A pathological study supported that the deposition of amyloid protein in CAA can be divided into three stages, from local vessels to the whole brain. In stage 1, CAA is restricted to leptomeningeal and cortical vessels of the neocortex. In stage 2, amyloid protein deposits in vessels of the neocortex, the allocortex, cerebellum, and midbrain. In stage 3, CAA-affected vessels are seen in all areas already involved in stage 2 and within the lower brainstem, the basal ganglia, and the thalamus [19].

SS is also an important imaging for CAA-related inflammation, which is considered as the inflammatory form of CAA, with patchy or confluent T2 hyperintensity in MRI, which is usually asymmetric [20,21]. A recent study showed that SS progression might be a potential biomarker for assessing disease severity and future ICH [22]. From the asymmetrical progress of ICH in three cases with diffused SS, we speculated that severer deposition of amyloid protein might be associated with the side of the severer white matter lesion and ICH. Amyloid protein may not deposit homogeneity in all the leptomeningeal vessels at early stage and become more serious in some local leptomeningeal vessels in one side, which causes more severe LA, SS, and ICH on the same side. However, in the late phase when amyloid protein widely spreads to the whole brain vessels, the distribution of lobar hemorrhage becomes more widely spread and unpredictable.

Our preliminary findings suggested that even the recurrence of ICH was not in the same place of SS, which had strong association with it. The early recurrence of ICH might be on the same side of SS. These findings were consistent with the recent follow-up study [23]. This may provide additional insights into the mechanisms of ICH recurrence in patients with CAA. Because of the limited number of clinical follow-up cases, further research in combination with pathology and multi-center follow-up studies is needed.
